# Visceral Leishmaniasis in Southeastern Iran: A Narrative Review

**Published:** 2017

**Authors:** Iraj SHARIFI, Mohammad Reza AFLATOONIAN, Mohammad Hossein DAEI PARIZI, Ali HOSSEININASAB, Mahshid MOSTAFAVI, Mehdi BAMOROVAT, Abass AGHAEI AFSHAR, Mehdi MOHEBALI, Hossein KESHAVARZ, Hamid DANESHVAR, Zahra BABAEI, Hossein MAHMOUDVAND, Mohammad Ali MOHAMMADI, Fatemeh SHARIFI, Mohammad BARATI, Hossein KAMIABI, Tabandeh KHALEGHI

**Affiliations:** 1. Leishmaniasis Research Center, Kerman University of Medical Sciences, Kerman, Iran; 2. Tropical and Infectious Diseases Research Center, Kerman University of Medical Sciences, Kerman, Iran; 3. Afzalipour Hospital, School of Medicine, Kerman University of Medical Sciences, Kerman, Iran; 4. Dept. of Medical Parasitology and Mycology, School of Public Health, Tehran University of Medical Sciences, Tehran, Iran; 5. Pharmaceutics Research Center, Institute of Neuropharmacology, Kerman University of Medical Sciences, Kerman, Iran; 6. Dept. of Medical Parasitology and Mycology, School of Medicine, AJA University of Medical Sciences, Tehran, Iran

**Keywords:** Visceral leishmaniasis, Kala-azar, *Leishmania infantum*, Iran

## Abstract

**Background:**

Visceral leishmaniasis (VL) has strong links with poverty, substantial medical and veterinary impacts. This review aimed to focus in studies published during 1994–2016 on VL in southeastern Iran.

**Methods:**

The present review is based on expert knowledge and historical studies published during the past 23 yr (1994–2016) on VL in southeastern Iran. In addition, related literature found in PubMed by using the keywords such as visceral leishmaniasis, kala-azar, and *Leishmania infantum* are included.

**Results:**

Overall, 118 children aged 4.2 yr were detected as infected with human VL (HVL). The majority of the cases were from Orzoieh district (37.1%) in southwest of Kerman Province, followed by Sirjan (15.7%), Jiroft (14.8%), Kahnuj (9.3%) and to lesser extent from other areas. The male to female ratio was 1.7. The three most frequent clinical features were represented by fever (100.0%), anemia (95.0%) and splenomegaly (91.5%). Altogether, 42.0% of the VL cases developed secondary bacterial infections, the overall case-fatality rate was 3.4%, and majorities (88.0%) of the VL patients were undernourished. Overall, 733 dogs and wild canines were examined by different techniques with various seroprevalence ranges.

**Conclusion:**

In southeastern Iran, VL is endemic in Orzoieh district in Kerman Province. While the dogs are implicated as the main domestic reservoir of VL, wide range of wild canines can serve as a secondary potential reservoir host.

## Introduction

Leishmaniasis represents a group of parasitic disease with complex spectrum of clinical syndromes caused by the species of Genus *Leishmania.* The clinical manifestations of the disease vary significantly but are often classified into three distinct clinical forms including of cutaneous leishmaniasis (CL), mucocutaneous leishmaniasis (MCL) and visceral leishmaniasis (VL) (1, 2). VL also known as kala–azar is the most severe and fatal form of leishmaniasis, widely distributed across the globe, mainly in tropical and sub-tropical countries (3).

This disease poses a serious clinical and veterinary impact in terms of morbidity and mortality. An estimate of 0.2 to 0.4 million cases suffer from potentially fatal VL, per year (4). Kala-azar is one of the most neglected and vector-borne diseases which has strong links with poverty exerting major impact and substantial health problems among the poor (5) and up to 40000 deaths per annum (4). Approximately, 90% of all VL cases occur in Indian subcontinent, East Africa and Brazil (4, 6).

Many factors play important roles in distribution of VL cases in each country and region including climatic condition, environmental changes, inadequate living conditions, malnourishment, presence of suitable reservoir hosts and principal biological vectors (7, 8). VL presents itself with various clinical features. It is often accompanied by other conditions leading to a fatality rate of 5%–15% and up to 95% if treatment is delayed or ignored (3). At present, the overall number of VL cases are rising globally, due to lack of efficacious vaccine, difficulty in controlling wide range of reservoir hosts and diverse phlebotomine sand fly species and emergence of resistance against drugs of choice (9).

In Iran, majority of VL cases are reported from two main endemic foci consisting of northwestern (East Azerbaijan and Ardabil provinces) and southern areas (Fars Province), and more recently to lesser extent from Bushehr, Qom, Kerman, northern Khorasan and other parts of the country (10–19). Over the past years, there has been an average of 100–300 new cases of VL reported annually (15). Most of the patients have been registered in remote areas, notably from rural and nomadic regions where living standards are poor and people dwell in close contact with the main animal reservoir, dogs (*Canis familiaris*). The causative agent is *L. infantum,* and over 90% of the cases have been found in children of up to 12 years of age (3,15).

In recent years, the incidence of Mediterranean VL caused by *L. infantum* has been decreasing in many foci worldwide and in Iran, mainly due to improvement of hygienic conditions and promotion of living standards (9). This is in contrast with kala-azar cases caused by *L. donovani,* which continues to rise and kill thousands of individuals each year in Indian Subcontinent and African countries (20).

The prevalence of canine VL (CVL) in endemic areas of Iran ranges between 14.2% and 17.4% (10, 21–27), although this range varies depending upon the geographical location, the method performed in the detection of CVL and also the availability of the major domestic and wild canine reservoir host and presence of specific species of vector (7, 8).

## Methods

### Literature search and Scope

The present review is based on expert knowledge and historical studies published during the past 23 yr (1994–2016) on VL in southeastern Iran. In addition, related literature found in PubMed by using the keywords such as visceral leishmaniasis, kala-azar, and *Leishmania infantum* are included. A review of other literature has been performed and almost all existing registered cases and reports have been presented. Issues such as clinical manifestations, epidemiological features, diagnostic methods, current treatment regimens and vector incrimination have been presented and discussed.

So far, no comprehensive review study has been published on VL from southeastern Iran. Therefore, presenting this narrative review work could help in better understanding of the clinical and veterinary impacts of the VL disease for planning possible future control strategies.

### Study area

**Fig. 1:**
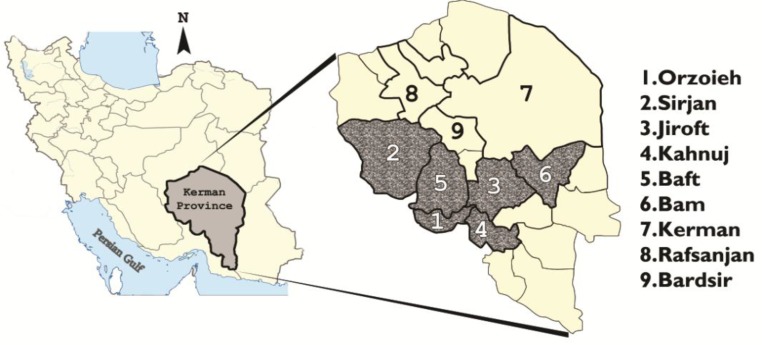
Spatial distribution of visceral leishmaniasis cases by districts in southeastern Iran

Province of Kerman covers 180726 km^2^ areas with approximately three million populations; located 1080 km from Tehran in southeastern Iran. It is the largest province of the country, representing 11.0% of the total land in 24 districts. An estimated 60% (1850000 individuals) of the population live as nomadic and rural life, generally in remote areas of the Province, more frequently in south and southwest. Although, attempt has been made to settle the nomad tribes’ population (nearly 100000 individuals) but they still travel back and forth through a distinct route from south to Kahnuj with altitude of 300 m above the sea level toward Jiroft, Orzoieh, Baft, Sirjon and Hajiabad, 1500 m above the sea level in mountainous areas toward north. Approximately, over 90.0% of the kala-azar cases belong to Orzoieh, Sirjan, Jiroft, Kahnuj, Baft, Bam and Kerman districts (Fig. 1). The Province possesses 25.0% of the total gardens including orchard of oranges, pistachios, palm trees, and walnuts in the country. A considerable number of population, more commonly from Sistan and Bluchestan and from neighboring cities of Hormozgan Provinces for easiness of accessibility seek medical treatment in Kerman Province; although, a similar attention is provided vice versa.

## Results

### Clinical manifestations Human

In general, the clinical manifestations of VL are similar in all endemic areas. Such clinical picture of VL is often indistinguishable from other infectious diseases. In acute cases, there may be an abrupt onset of high fever and chills that often mimics other infectious microbial diseases (28–30). The fever pattern typically shows two peaks per day, often used as a useful tool for clinical diagnosis (31). In chronic course, there is an insidious onset of fever, loss of appetite, weight loss, hepatosplenomegaly, anemia and pancytopenia causing abdominal distention and weakness. In endemic areas, low-grade symptoms may persist for few weeks to several months before progressing to fully developed VL manifestations, because these symptoms are relatively well tolerated and usually are not severe to seek medical attentions. The clinical picture of VL is multifactorial. Despite the complexity, *Leishmania* biology, genetic nature, host immunology, and physiology are major determinants of clinical outcome (8).

### Dogs

Reticuloendothelial system (RES) is the most affected organs, with zoonotic visceral leishmaniasis (ZVL), characterized by an acute or chronic and proliferative inflammatory process. The broad spectrum of clinical syndrome depends on the stage of the infection. Presentations, such as alopecia around the eyes and ears, onychogryphosis, weight loss, hepatosplenomegaly, lymphadenopathy muscular weakness, anemia, renal failure and followed by cachexia are typical manifestations (22, 27).

### Diagnostic methods

The diagnosis of VL is complicated by the fact that this disease shares its clinical manifestations with other parasitic and microbial infections.

### Parasitological methods

Definitive diagnosis relies on demonstration of the parasites, amastigotes (Leishman bodies) in the bone marrow or in other tissues. In both human and principal reservoir host, dogs and wild canines, spleen puncture is definitely an effective method for detecting infection, but it is a risky task and may result in massive bleeding (3, 32). Liver puncture is much safer but is not productive. In routine medical practice, sternum bone marrow or iliac crest aspiration detects the organisms and is generally considered the method of choice; although, the sensitivity is low. In some occasion, buffy coat films, prepared from peripheral venous blood, could be used for demonstrations of amastigotes in mononuclear phagocyte cells, but with low sensitivity.

### Culturing and animal inoculation

Whenever possible, culturing of materials obtained from RES, using biphasic, medium (NNN) and further subculturing into monophasic culture media such as RPMI1640 and maintaining at ambient temperature, 22 °C–26 °C, could be used (3). However, in general, because of low infection rate, the chance of growth is negligible and often inconclusive. Touch smear preparation of spleen spices followed by methanol fixation and Giemsa staining could reveal amastigotes by high dry microscopy. For research purposes, intraperitoneal inoculation of golden hamsters, a standard biological method of choice, might be performed.

### Serological methods

A number of serological techniques using different antigens and assays are available. Currently, ELISA, indirect fluorescent antibody test (IFAT), direct agglutination test (DAT) and rK39 (recombinant K39 antigen) are widely used tests and have demonstrated excellent sensitivity and specificity for the diagnosis of VL (17, 33–35).

### Molecular methods

For identification and characterization of the causative agent to species and strain level, molecular techniques employing DNA-based methods, more frequently PCR techniques followed by sequencing are routinely used (35, 36).

### Vector incrimination

Interestingly, in the main endemic areas in Orzoieh district in the Province of Kerman (south to the Baft district) two *Leishmania* species coexist; *L.major*, the cause of zoonotic cutaneous leishmaniasis (ZCL) and *L.infantum*, the causative agent of kala-azar. Only one study has been performed by sticky traps and aspiratory methods in the closely related localities within the district (37). The predominant sand fly species were *Phlebotomus papatasi* (33.7%) and *P. alexandri* (29.8%) during the peak activity of the sand fly species. Based on the results of blood-fed index by ELISA test and using dog and human antisera, they were positive for human blood. At present, the role of *P. alexandri* as being implicated in the transmission of *L. infantum* in the area is not well clear. However, due to high index of human blood feeding, it is assumed that this species might be a probable vector for kala-azar in this locality. *P. alexandri* species has been implicated as principal phlebotomine sand fly vector of kala-azar in some parts of the world including China (38).

### Current therapy

For many years, pentavalent antimony drugs particularly meglumine antimoniate (Glucan-time®, Sanofi-Aventis, France) have been used as the first-line drugs to treat human VL nationally and also in Kerman Province (39). The recommended regimen is 50 mg /kg/day of Glucantime® for 3–4 wk. The drug is given intramuscularly (IM). In some instances, amphotericin B has been used in patients with compromised immune system.

### Epidemiology

A total of 118 children aged 4.2 yr (range; 0.2–13 yr) comprising 75 boys (63.6%) and 43 girls (36.4%) were diagnosed and confirmed by combination of parasitological, serological and molecular methods as HVL patients between 1994 and 2011 in southeastern Iran (Table 1). The majority of the cases were from Orzoieh district in southwest of Kerman Province (37.1%), followed by Sirjan (15.7%), Jiroft (14.8%), Kahnuj (9.3%), Hajiabad in Hormozgan Province (7.4%), Baft (6.5%), Bam and Kerman (3.7%, each) and Rafsanjan and Bardsir (0.9%, each) (Table 2). In Mediterranean kala-azar, the proportion of male to female is significant mainly due to higher exposure of boys to the source of infection and incomplete dressing.

**Table 1:** Reported cases of human visceral leishmaniasis in Kerman Province, southeastern Iran, 1981–2011

**Cases(No)**	**Mean age (yr)**	**Reported years**	**Diagnostic Method**	**Reference**
**40**	4.2(0.5–12)	1981–1992	BMA	32
**68**	4.3(0.5–13)	1993–2006	BMA	29
**10**	3.5(0.2–7)	2007–2011	BMA and nested PCR	44

BMA; bone marrow aspiration

**Table 2:** Distribution of human visceral leishmaniasis cases by district, southeastern Iran

**Orzoieh**	**Sirjan**	**Jiroft**	**Kahnuj**	**Hajiabad**
**37.1%**	157%	14.8%	9.3%	74%
**Baft**	**Bam**	**Kerman**	**Rafsanjani**	**Bardsir**
**6.5%**	3.7%	3.7%	0.9%	0.9%

Various clinical presentations are given in Table 3. As the time passes, infiltration of infected white blood cells particularly mononuclear phagocytes and proliferation of these cells, frequently in RES such as spleen, liver, lymph nodes and bone marrow results in massive enlargement of these organs (29). Bone marrow cells become infected with amastigotes and patients develop pancytopenia (depression of erythrocytes, leukocytes, and platelets) and eventually suppression of immune responses (30), which make kala-azar patients susceptible to microbial super-infections. Therefore, 42.0% of the patients developed secondary bacterial co-infections of the urinary tract, blood respiratory and gastro-intestinal tracts and skin as presented in Table 4. Unfortunately, such underlying infections may complicate the syndrome, produce unusual clinical features (29, 40, 41) and persist for weeks to several months (average 32 d in this review before visiting a physician).

**Table 3:** Clinical manifestations accompanying human visceral leishmaniasis, southeastern Iran

**Fever**	**Anemia**	**Splenomegaly**	**Hepatomegaly**	**Anorexia**
**100.0%**	95.5%	91.5%	77.1%	47.4%
**Paleness**	**Weight loss**	**Dysentery/Diarrhea**	**Chills**	**Hemorrhage**
**46.2%**	24.4%	23.1%	14.7%	5.9%

**Table 4:** Secondary bacterial coninfections with visceral leishmaniasis in children, southeastern Iran

**Site**	**Children (%)**	**Etiology**
Urinary tract	36	*Escherichia coli*
		*Enterobacter* spp.
		*Klebsiella* spp.
		*Proteus* spp.
Blood (Septicemia)	28	*Staphylococcus aureus*
		coagulase-negative
		*Pseudomnas* spp.
Respiratory tract	16	ND
Gastro-intestinal tract	12	*Shigella* spp.
		*E.coli*
Skin	8	*S. aureus*

ND; not determined. Table 4 is taken from reference 41

Seroprevalence of HVL infection showed different ranges at different time intervals depending upon the tests performed, spatial distribution and selection of samples in southeastern Iran (Table 5). It varied sharply among the districts.

**Table 5:** Seroprevalence of human visceral infection in southeastern Iran

**District**	**Examined No.**	**Method performed**	**Seroprevalence No. (%)**	**Reference**
Baft 1994	1304	IFAT	39 (3.0)	34
Jiroft	950	IFAT	18 (1.9)	17
2000–2001	950	ELISA	171 (18.3)	17
Orzoieh 2009–2010	1476	DAT	14 (0.95)	16

Mortality data were considerably sparse and limited generally to hospital-based deaths, only. The overall case-fatality rate was four patients (3.4%) out of 118 cases of children. Most of the patients were poor and majority from rural and nomadic tribes (83.0%) in remote areas of the country, who are not able to seek medical treatment at the earliest time. The majority (88.0%) of the HVL patients were undernourished with moderate to severe malnourishment (29). Some patients, due to passive case detection approaches go underreported and the degree of unnoticed HVL cases in official surveillance data is not clearly known, but it varies country to country. It ranges between 1.3–1.7 fold in Brazil (42) and 4–8 folds in India (43), which are lower than the actual incidence rate found by active case detection strategies.

Evaluation of drug efficacy indicated that most of the cases responded well to meglumine antimoniate (Glucantime®) and seemed to be successful in treatment of patients. However, few cases (1.7%) had relapsed to the initial course of therapy treated with the drug (Glucantime®), consequently. This rate is much less than the range (10%) previously reported from other countries (3, 9). Similarly, in one case, with viscerotropic strains of *L. tropica* (the cause of anthroponotic cutaneous leishmaniasis), the patient was unresponsive, to Glucantime (44).

Although dogs have been implicated as the major domestic reservoir of *L. infantum,* two dogs of the total 471 stray dogs were infected with *L. tropica*, presenting typical manifestations resembling VL affected dogs due to *L. infantum* (45). Combination of parasitological, molecular, serological and biological methods detected significant numbers of asymptomatic infections and could help properly in discrimination between *L. infantum* and *L. tropica* (22). A case of viscerotopic manifestions due to *L. tropica* was found in a 5 yr old child who was unresponsive to conventional dosages of Glucantime® (44).

Prevalence of CVL infection by different tests is presented in Table 6. Most of the studies were performed from the districts in which HVL cases were reported (21–23, 26, 35, 46). Overall, 733 dogs (domestic and stray) and wild canids (jackals and foxes) were examined by various techniques. CVL infection was officially first reported in 1994 (46). VL was endemic in Orzoieh district and at that time Orzoieh was a part of Baft district, where currently it is segregated in form of separate district.

**Table 6:** Seroprevalence and molecular identification of canine visceral infection in southeastern Iran

**District**	**Animal**	**Examined (No.)**	**Seroprevalence No. (%)**	**Method Performed**	**Reference**
Orzoieh 1994	Domestic dogs	83	15(18.0) 12(14.5)	IFAT	51
	Domestic dogs Domestic dogs	83	1(1.2)	ELISA	51
		83	8(25.0)	BMA	51
	Jackals	32	7(22.0)	IFAT	51
		32	1(9.0)	ELISA	51
	Foxes	11	2(18.0)	IFAT	51
		11		ELISA	51
Orzoieh 2009–2010	Domestic dogs Domestic dogs	30	7(23.0)	DAT & Nested-PCR	16
		30	5(16.7)		16
Orzoieh 2009–2011	Stray dogs	32	6(18.8)	ELISA	26
Orzoieh 2013–2014	Homestead dogs	59	18(30.5) 22(37.3)	IFAT	23
		59		Nested-PCR	23
Bam 2009–2011	Stray dogs	57	13(22.8)	ELISA	26
Zabol 2009–2011	Stray dogs	45	7(15.6)	ELISA	26
Kerman 2009–2011	Stray dogs	67	5(7.5)	ELISA	26
Kerman 2011	Domestic dogs	128	9(7.0)	ELISA	21
Kerman 2009–2010	Stray dogs	80	9(11.3)	DAT	22
		9	2(22.2)*	PCR	22
Jiroft 2013–2014	Domestic dogs	100	14(14.0)	ELISA	27
		18	17(94.4)	Nested-PCR	27

IFAT; indirect fluorescent antibody test, ELISA; enzyme-linked immunoabsorbent assay, BMA; bone marrow aspiration; DAT; direct agglutination test.

* Of two VL dogs, one was coinfected with *L. tropica.*

Various seroprevalence ranges were reported in dogs, depending on the location and type of the test performed (21–22, 26, 27, 46). While the seroprevalence was the lowest (7.0%) by ELISA in Kerman district, it was the highest by IFAT or nested – PCR in Orzoieh district. In general, there was no significant difference between male and female dogs, while the VL infection was significantly higher in older age group than younger ones. In addition to dogs, as principal and peridomestic reservoir of VL, a wide range of wild canids such as Jackals and foxes are found near human habitations (25, 46).

## Discussions

The clinical features of HVL in this location were considerably similar to those patients from other parts of the country and even the world (3, 40). Overall, 3.4% died due to poverty, undernourishment, seeking late medical treatment and secondary bacterial superinfections. The death rate was significantly lower than the tentative estimates of HVL deaths in global range (3). In rare cases viscerotopic *L. tropica* might cause VL, presenting typical manifestations. These cases are usually unresponsive to conventional therapy by Glucantime. Physicians should be aware of such clinical presentation in order to manage the patient properly. Similar viscerotropic syndrome due to *L. tropica* was reported among American troops who participated in Operation Desert Storm (47).

Dogs are the main reservoir host for dissemination of infection to human. In principal reservoir, canines and less well-adapted hosts including humans, VL can produce a wide range of manifestations including asymptomatic condition to very serious visceral form. Asymptomatic dogs and humans could constitute a major source of infection for the vector and in turn transmission to humans (31, 48–51), that is why dogs represent a significant public health threat to humans. A high proportion of seropositive dogs (over 50%) is asymptomatic and may propagate infection with no clinical manifestations for several years or even throughout life (25, 50). Moreover, such asymptomatic dogs are as infective to the vector as symptomatic ones (52). Transmission of VL infection among wildlife, domestic animals, and human is of great concern to public health. They can harbor the infection and serve as secondary potential reservoirs for transmission of the disease (53).

## Conclusion

1. Clinical and epidemiological data on the spatial distribution and prevalence of HVL and CVL are essential for planning and implementing appropriate control strategies.
2. VL in Orzoieh district is endemic, while it is sporadic to some extent in other districts in southeastern Iran.
3. Early detection of HVL cases, prompt treatment and integrated vector management by active and passive case detection approaches through an effective health surveillance system should receive high priority.
4. Since stray and feral dogs pose serious human and welfare problems, veterinary services should play a leading role in dog population control; to improve health and welfare of owned and stray dog population and to reduce numbers of stray dogs to an acceptable level.
5. A combination of applicable parasitological, serological and molecular methods are curtailed for the detection of the extent of infection in human and dogs in endemic areas, particularly those with latent and/or apparent manifestations in order to understand their role in epidemiology of ZVL.
6. Detection and surveillance of VL status in immunocompromised patients should receive special attention in future planning.
